# Tumor metabolism regulating chemosensitivity in ovarian cancer

**DOI:** 10.18632/genesandcancer.176

**Published:** 2018-05

**Authors:** Chae Young Han, David A. Patten, Richard B. Richardson, Mary-Ellen Harper, Benjamin K. Tsang

**Affiliations:** ^1^ Department of Obstetrics and Gynecology and Cellular and Molecular Medicine, University of Ottawa, and Chronic Disease Program, Ottawa Hospital Research Institute, Ottawa, Ontario, Canada; ^2^ State Key Laboratory of Quality Research in Chinese Medicine, Macau Institute for Applied Research in Medicine and Health, Macau University of Science and Technology, Avenida Wai Long, Taipa, Macao, China; ^3^ Canadian Nuclear Laboratories (CNL), Radiobiology and Health Branch, Chalk River Laboratories, Chalk River, Ontario, Canada; ^4^ Department of Biochemistry, Microbiology and Immunology, Faculty of Medicine, University of Ottawa, Ottawa, Canada

**Keywords:** ovarian cancer, chemoresistance, tumor metabolism, hexokinase 2, p53

## Abstract

Elevated metabolism is a key hallmark of multiple cancers, serving to fulfill high anabolic demands. Ovarian cancer (OVCA) is the fifth leading cause of cancer deaths in women with a high mortality rate (45%). Chemoresistance is a major hurdle for OVCA treatment. Although substantial evidence suggests that metabolic reprogramming contributes to anti-apoptosis and the metastasis of multiple cancers, the link between tumor metabolism and chemoresistance in OVCA remains unknown. While clinical trials targeting metabolic reprogramming alone have been met with limited success, the synergistic effect of inhibiting tumor-specific metabolism with traditional chemotherapy warrants further examination, particularly in OVCA. This review summarizes the role of key glycolytic enzymes and other metabolic synthesis pathways in the progression of cancer and chemoresistance in OVCA. Within this context, mitochondrial dynamics (fission, fusion and cristae structure) are addressed regarding their roles in controlling metabolism and apoptosis, closely associated with chemosensitivity. The roles of multiple key oncogenes (Akt, HIF-1α) and tumor suppressors (p53, PTEN) in metabolic regulation are also described. Next, this review summarizes recent research of metabolism and future direction. Finally, we examine clinical drugs and inhibitors to target glycolytic metabolism, as well as the rationale for such strategies as potential therapeutics to overcome chemoresistant OVCA.

## I INTRODUCTION

Ovarian cancer (OVCA) is the fifth leading cause of cancer deaths in women and has a high mortality rate (30-50%) [1, 2]. Late diagnosis and the development of chemoresistance are major hurdles to successful therapy. Due to non-specific clinical symptoms and the absence of early biomarkers, OVCA is usually diagnosed in advanced stages. Epithelial OVCA accounts for more than 90% of incidences and has the highest mortality rates [3]. Within epithelial OVCA, high-grade serous is most frequently found (70%) among different sub-types (high-grade, low-grade, endometroid, clear cell, and mucinous) [2]. The standard treatment of OVCA is debulking surgery followed by chemotherapy, but 70% of patients in advanced stage experience chemoresistance or relapse within 15 months following treatment [4].

Cis-diamminedichloroplatinum (II) (Cisplatin, CDDP) and its analogues (e.g., Carboplatin and Oxaliplatin) are commonly used platinum containing anti-cancer drugs as DNA damaging agents in OVCA. The underlying mechanisms of chemoresistance are multifactorial, partly due to defects in apoptosis, dysregulation of an oncogene, defects in tumor suppressors, and increased metabolism [5, 6]. The master tumor suppressor p53 is responsible for apoptosis of cancer cells and is critical to the chemoresponsiveness of OVCA [7, 8].

*TP53* mutations occur in 70% cases of OVCA and in more than 90% cases in high-grade serous epithelial OVCA [9], and is closely associated with chemoresistance [10]. Understanding in-depth metabolic and molecular mechanisms are required in the pursuit for effective therapeutic targets to overcome chemoresistant OVCA. Metabolic reprogramming enables cancer cells to fulfill their high proliferation and survival potentials [11]. Since Otto Warburg proposed a high rate of aerobic glycolysis in cancer cells (Warburg effect) in 1923 [12, 13], the unique metabolic character of cancer cells has been intensely investigated as a target for cancer therapy. The Warburg effect specifically proposes that glycolysis is a main metabolic pathway for ATP generation and oxidative phosphorylation (OXPHOS) is impaired in cancer cells; however, recent research has demonstrated that both pathways are relatively elevated in cancer cells compared to normal cells [11, 14]. Moreover, it may be that the ability of cancer cells to switch between energy substrates and metabolic pathways (termed bioenergetic flexibility) is associated with poor prognosis, including metastasis [15, 16]. Glycolysis works as precursor pathway, since its metabolites and products are required for downstream pathways including: the tricarboxylic acid (TCA) cycle and OXPHOS; pentose phosphate pathway (PPP) for ribonucleotide synthesis and Nicotinamide adenine dinucleotide phosphate (NADPH); glycosylation and gluconeogenesis; amino acid biosynthesis, and fatty acid synthesis [11, 17]. In addition to glycolysis, the PPP pathway is highly elevated in cancer [18, 19], providing anabolic substrates for cancer growth and reductive intermediates (e.g., NADPH) for glutathione (GSH) synthesis and protection from oxidative damage.

Fatty acid metabolism is similarly altered in cancer. Unsaturated lipids are increased in OVCA [20, 21], which induces stemness, whereas lipid desaturation impairs cancer stemness and tumor initiation [21]. Cellular glycogen accumulation is observed as a feature of clear cell, a sub-type of epithelial OVCA, which frequently develops chemoresistance [22]. Also, glycogen accumulation is elevated in hypoxic conditions, a core component of the solid tumor microenvironment [22, 23]. These findings suggest that specific metabolic phenotypes enhance chemoresistance in OVCA.

**Figure 1:**
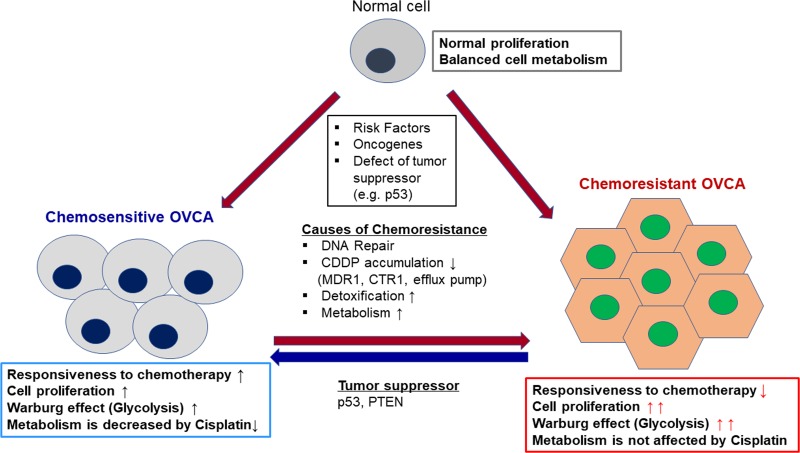
Reprogramming tumor metabolism in chemoresistant OVCA Metabolic reprogramming of cancer cells contributes to their transformation. The Warburg effect (see main text) often allows cancerous cells to maintain energy production in otherwise energy poor conditions. Genetic and environmental factors may transform normal cells to either chemosensitive or chemoresistant OVCA. However, the majority of chemoresistant cells stem from chemosensitive cancer cells that acquire their resistance due to multiple factors: increased DNA Repair, CDDP detoxification, increased metabolism, and the upregulation of multi-drug resistance and copper transporters. As a result, chemoresistant cells have markedly higher rates of proliferation, and their metabolism is less sensitive to bouts of chemotherapeutics. We and other, have also demonstrated that the recovery of defective p53 and PTEN can sensitize chemoresistant cells to chemotherapy.

However, it remains unclear whether metabolic reprogramming in cancer is associated with chemoresistance and what therapeutic approaches could modulate metabolic phenotypes associated with chemoresistance of OVCA (Figure 1). In this review, we discuss: 1) the cellular and molecular mechanisms involved in metabolic reprogramming; 2) the influence of metabolism on apoptosis and the related functions of mitochondria; 3) whether and how oncogenes/tumor suppressors regulate the role of key glycolysis enzymes; 4) future strategies for targeting metabolism in cancer treatment, including combining such therapies with traditional chemotherapeutics; and lastly, 5) the importance of tumor microenvironment and potential novel metabolic strategies.

## II THE ROLE OF GLYCOLYTIC METABOLISM IN CHEMORESISTANCE

**Figure 2:**
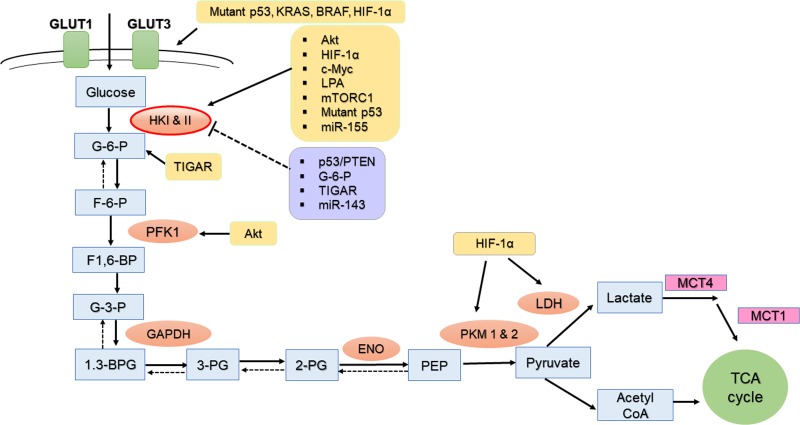
The regulation of HKII and other glycolytic enzymes in ovarian cancer cells Enhanced glycolysis in cancer cells, through a combination of metabolic pathways, drives glucose utilization to fulfill high anabolic demands. In this schematic diagram of the glycolysis pathway, metabolites are shown as square boxes. Key regulatory molecules which either promote (yellow box) or suppresses (purple box) glycolytic enzymes and metabolites such as G-6-P are shown. GLUT (glucose transporter), G-6-P (glucose-6-phosphate), F-6-P (fructose-6-phosphate), F-1,6-BP (fructose-1,6-bisphosphate), G-3-P( glyceraldehyde 3-phosphate), 1,3-BPG (1,3-bisphosphoglycerate), 2-PG (2-phosphoglycerate), 3-PG (3-phophosphoglycerate); PEP (phosphoenolpyruvate), PFK (phosphofructokinase), GAPDH (glyceraldehyde 3-phosphate dehydrogenase), TIGAR (TP53-inducible glycolysis and apoptosis regulator), ENO (enolase), MCT (monocarboxylate transporter). mTORC1 (mTOR complex 1), Mutant p53 (defect p53), PFKFB (6‑phosphofructo 2‑kinase/fructose‑2,6‑bisphosphatase), Akt (Protein Kinase B), HIF-1α (hypoxia Inducible Factor-1α), c-Myc (v-myc avian myelocytomatosis viral oncogene homolog), and LPA (Lysophosphatidic acid).

Accelerated glycolysis is a key hallmark of tumorigenesis [12, 24, 25]. Glycolysis is the first metabolic pathway converting glucose to pyruvate. Beyond their metabolic functions, key glycolytic enzymes have been shown to enhance the survival and progression of tumors associated with drug resistance (Figure 2). Metabolic adaptation of excessive glycolysis in cancer is mediated by hyperactive glycolytic enzymes, and this is primarily due to dysregulation of tumor suppressors or activation of oncogenes [26].

### Glucose transporters

Glucose transporters (GLUTs) are a large group of membrane transport proteins that facilitate the transport of glucose over a plasma membrane [27]. There are 14 identified GLUTs (SCL2A1 to SCL2A14). Among them, GLUT1 has been identified as a hypoxic marker and plays the critical role in the Warburg effect [28]. In cancer cells, GLUT1 is highly expressed and facilitates metastasis of tumor and poor prognosis in multiple cancers including lung, stomach, breast, and kidney [28, 29]. High level of GLUT1 expression is observed in membrane of high-grade serous OVCA, whereas GLUT1 expression is negatively correlated with fasting glucose concentration, explaining its glucose uptalking [30]. In OVCA, by modulating GLUT1 membrane trafficking, phytochemicals such as resveratrol decreases glycolysis and induces apoptosis [31].

### Hexokinase II

Hexokinase (HK) is the enzyme involved in the first committed and irreversible step in glycolysis, converting glucose to glucose-6-phosphate (G-6-P). There are five HK isoforms including recently discovered HKV (HKI, II, III, IV, and V) [32]. HKI to HKII have high substrate affinity, while HKIV has low glucose affinity and serves as an important glucose sensor (e.g., to control β-cell insulin release). A fifth HK as novel isoform was recently found, and it remains to be further explored [32]. HKI and HKII are found in many cellular compartments, including being bound to the outer mitochondrial membrane (OMM), and localized within mitochondria [33]. Both HKI and HKII are inhibited by G-6-P, its catalytic product *via* feedback inhibition [13]. HKII, a predominant isoform in insulin sensitive tissues (adipose, skeletal, and cardiac muscles), is highly expressed in multiple tumors [34]. Recent research demonstrates that HKII is highly associated with tumorigenesis and cancer cell survival [25, 35]. In mouse models, deletion of HKII significantly decreased tumor burden and prolonged survival, suggesting that HKII is a critical factor involved in tumor progression [36]. Inhibition of HKII restored normal glycolysis and OXPHOS pathway as well as mitochondrial biogenesis in glioblastoma [37]. HKII depletion also increased intrinsic apoptosis by increased mitochondrial permeability in chemoresistant brain cancer cells [37]. Lysophosphatidic acid (LPA), a lipid growth factor and G protein-coupled receptor (GPCR) ligand are significantly increased in OVCA, triggering the activation of hypoxia inducible factor-1α (HIF-1α) and inducing GLUT1 and HKII, thus shifting cells towards a glycolytic metabolism [38]. Zhang et al. have demonstrated that HKII contributes to chemoresistance by enhancing CDDP-induced extracellular signal-regulated kinases (ERK) 1/2 phosphorylation and autophagy [39].

### Phosphofructokinase 1 (PFK1)

Phosphofructokinase (PFK) 1 is responsible for converting fructose-6-phosphate (F-6-P) to fructose 1,6 bisphosphate (F-1,6-BP), the second irreversible step in glycolysis [40, 41]. Somatic mutation of PFK and altered structure promote altered glycolytic flux in cancer [42]. The most potent allosteric activator of PFK is fructose-2,6-bisphosphate (F-2,6-BP) and its synthesis and degradation depend on the 6-phosphofructo-2-kinase/fructose-2,6-bisphosphatases (PFKFB) [43]. PFKFBs phosphorylate F-6-P to F-2,6-BP, which in turn activates PFK1, forwarding glycolytic flux to lactate. Among the five PFKFB isoenzymes (PFKFB 1-4, and TIGAR), PFKFB3 has the highest kinase/phosphate activity ratio, leading to elevated F-2,6-BPase and resulting in high glycolysis rate. PFKFB3 promotes cell cycle progression and suppresses apoptosis by facilitating cyclin dependent kinase (Cdk)-induced degradation of p27, a key apoptotic activator [40]. Its product, F-2,6-BP is shown to be highly expressed in ovarian and breast cancers [43]. Conversely, PFKFB4 depletion increased apoptosis and the production of reactive oxygen species (ROS) in mitotically arrested cells. It also significantly enhanced mitotic cell death in OVCA with paclitaxel treatment [44].

### Pyruvate kinase

Pyruvate kinase (PK) catalyzes the last irreversible reaction of glycolysis, converting phosphoenolpyruvate to pyruvate. Two distinct PK genes, [*L/R* (in liver and red blood cells) and *PKM2* (in muscle)] encode four PK isoforms: PKL, PKR, PKM1, and PKM2. Among them, PKM2 is allosterically regulated by various metabolites [45, 46]. The activity of PKM2 enables cancer cells to adapt to altered tumor metabolic conditions. Phosphorylated PKM2 dimers stimulate a high rate of nucleotide and amino acid biosynthesis, while indirectly sustaining the Warburg effect [46]. Depletion of PKM2 in a breast cancer mouse xenograft model showed a reversal of the Warburg effect and inhibits tumor growth [47]. High expression of PKM2 is found in malignant OVCA, suggesting its late stage detection role of poor prognosis [48]. The association of PKM2 with tumorigenesis depends on the type of cancer, as the suppression of PKM2 did not show anti-tumor activity in breast and colon cancer, but did in leukemia [49, 50].

### Lactate dehydrogenase

Lactate dehydrogenase (LDH) catalyzes the interconversion of pyruvate and lactate, and of NADH and NAD+. Four LDH isozymes exist (LDH-A, LDH-B, LDH-C, and LDH-D), with LDH-A being predominantly expressed in the majority of tissues. Elevated levels of LDH-A are associated with multiple solid tumors and poor prognosis of cancer [51]. As LDH-A promotes the reduction of pyruvate to lactate for NADH production, whereas LDH-B favors the reverse reaction; LDH-A is a key enzyme involved in the Warburg effect. Depletion of LDH-A in tumor cells and in mice reduces tumor growth [52]. Recent research demonstrates that LDH supports enhanced rates of oxidative metabolism in tumors e.g., TCA cycle activity [53, 54]. Moreover, excessive activity of LDH in glycolysis can lead to local acidification of the tumor microenvironment, a favorable condition for tumor invasion and metastasis [55, 56]. High level of LDH is observed in serum and peritoneal fluid from OVCA patients compared with that of benign tumor, suggesting its possible role as potential prognostic biomarker [57, 58]. Lactate, the end product of LDH, a key gluconeogenic precursor, is moved intracellularly and intercellularly (e.g., Lactate shuttle) *via* monocarboxylate transporters MCT1 and MCT4, proposed by San-Millan et. al [59]. This notion is also supported by the evidence that high expression of MCT1 promotes the CDDP resistance by antagonizing the effect of first apoptosis of signal receptor (Fas) in epithelial OVCA [60]. High levels of lactate, as key elements for energy source and tumorigenesis, have been observed along with subsequent active glycolysis in many cancers [61].

## III THE INFLUENCE OF ELEVATED GLYCOLYSIS ON MITOCHONDRIAL-MEDIATED APOPTOSIS IN CANCER

### A The role of mitochondrial HKII for cell survival

**Figure 3:**
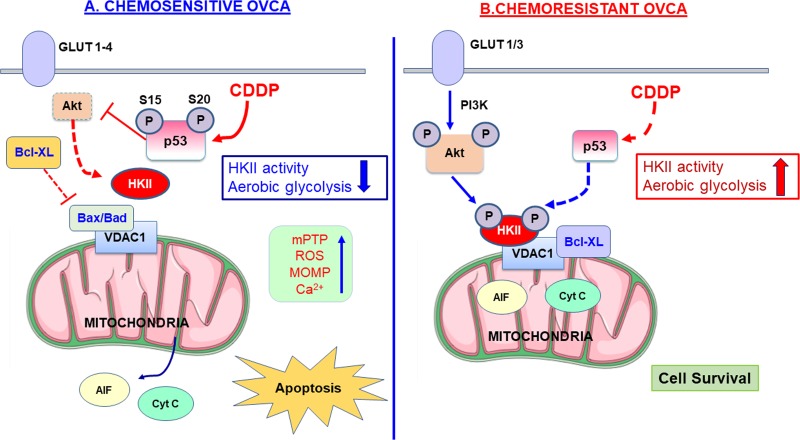
Mitochondrial-HKII drives chemoresistance in OVCA A hypothetical model demonstrating mechanisms regulating the mitochondrial localization of HK II and apoptosis. **A.** In chemosensitive OVCA cells, CDDP-induced detachment of mitochondrial bound HK II (mito-HKII) from VDAC is required for the induction of apoptosis. Chemotherapy such as CDDP induces the phosphorylation of p53 at Ser 15 (S15) and Ser 20 (S20), which suppresses phosphorylation of Akt, and promotes binding of HKII to mitochondria. In the absence of mito-HKII, proapoptotic Bcl-2 family proteins (Bax and Bad) interact with VDAC, increasing MPTP, ROS, MOMP, and Ca^2+^ in the OMM where AIF and cytochrome c are released. **B.** In chemoresistant OVCA cells, CDDP-induced apoptosis is attenuated due to defective p53. Through increased GLUT1 & 3 membrane trafficking, glucose is transported into the cell. Activated PI3-kinase phosphorylates lipids in the plasma membrane where Akt is recruited for activation. Activated Akt phosphorylates HKII (P-HKII), facilitates the translocation of Bcl-X_L_ and promotes binding of P-HKII to VDAC on the OMM, thus preventing apoptosis. Bcl-X_L_ directly interacts with VDAC, closing the mitochondrial ion channel and decreasing MOMP, while HKII inhibits apoptosis by competing for the binding sites for Bax and Bad of VDAC [26].

Intrinsic (mitochondria-mediated) apoptosis is initiated by the loss of mitochondrial membrane potential, increased mitochondrial outer membrane permeabilization (MOMP), and the subsequent release of mitochondrial death proteins including cytochrome c [62]. In addition to its role in glycolysis, HKII is involved in protecting mitochondria and the suppression of intrinsic apoptosis [63] (Figure 3), whereas its depletion of HKII enhances apoptosis and sensitivity to external stimuli. Ectopic expression of HKII protects lung cancer cells and renal epithelial cells from oxidative injury or cell death [64, 65]. Depletion of HKII in human HCC cells enforces metabolic pathway toward OXPHOS through maintaining TCA flux, thus facilitating cell death and inhibiting tumorigenesis [66].

In line with its role in regulating MOMP, HKI and HKII can bind to voltage-dependent anion channel 1 (VDAC1), a likely component of the mitochondrial permeability transition pore (mPTP) [25, 67]. Approximately 80% of HKII is localized with mitochondria in cancer cells [33]. Accumulating studies showed that HKII bound to mitochondria (mito-HKII) enhances tight coupling of glucose phosphorylation to mitochondrial ATP generation [68, 69]. DeWaal et al. showed that ectopic expression of mitochondrial binding-deficient HKII failed to restore cell proliferation and tumorigenesis, emphasizing pivotal role of mito-HKII [66]. Mitochondrial Ca^2+^ overload and ROS induce the opening of mPTP and consequent rupture. Mito-HKII provides cellular protection against this Ca^2+^ overload and mPTP opening [35].

The interaction of HKII with Bcl-2 members also affects apoptotic processes, particularly the execution of intrinsic apoptosis. Some anti-apoptotic molecules of Bcl-2 family (e.g., Bcl-2 and Bcl-xL) stay in the outer mitochondrial membrane (OMM), whereas pro-apoptotic members (Bad, Bax, and Bid) are translocated from the cytosol to the OMM upon initiation of apoptotic signaling [70, 71]. In mitochondrial-mediated cell death, activated Bax/Bak forms a pore at the OMM, induces MOMP, and results in the release of apoptotic factors such as cytochrome c from the inter-membrane space [71], which then accelerates the opening of VDAC [72]. Conversely, anti-apoptotic Bcl-2 family members, such as Bcl-xL interact with VDAC, inhibiting cytochrome c release by modulating OMM integrity or MOMP [70, 73]. Mito-HKII inhibits binding of Bax to Bcl-2 through competitive inhibition and antagonizes truncated Bid (tBid)-mediated apoptosis [74, 75]. Upon cell stress (e.g., glucose restriction or treatment with Metformin, Clotrimazole, Flavonoid-429, 2-DG, or 3-Bromopyruvate) HKII is detached from VDAC [76-79], inducing dysregulation of mitochondria by opening the mPTP, increasing the MOMP, and subsequent inducing of mitochondrial-mediated apoptosis [74, 80]. HKII dissociation from mitochondria using an artificial peptide also was shown to induce apoptosis without changing mitochondrial membrane potential, ROS production, or OXPHOS [78]. This suggests that detachment of HKII from mitochondria is critical for intrinsic apoptosis. However, the molecular mechanism of HKII detachment remains to be elucidated.

Cristae structural alterations and changes in the ultrastructure of the inner mitochondrial membrane compartments also significantly contribute to apoptotic signaling [81, 82]. Optic Atrophy 1 (OPA1) is a pro-fusion dynamin-related protein in the inner mitochondrial membrane that regulates cristae structure. OPA1 associates with itself to form oligomers, which act to maintain narrow cristae and narrow cristae junctions. However, Bax/Bak activation can disrupt OPA1 oligomerization, thereby widening cristae, and aiding in the release of pro-apoptotic factors and ultimately inducing apoptosis [82]. We observed that CDDP induced OPA1 processing and intact OPA1 is associated with chemoresistance [83, 84]. However, it remains unknown if and how mito-HKII is involved in Bax - OPA1 interaction and how this affects OPA1 oligomerization and cristae structure [81].

In addition, the role of uncoupling proteins (UCPs) in modulating mitochondrial HKII has been demonstrated. UCPs are mitochondrial anion transporters of the inner mitochondrial membrane and control the coupling efficiency of OXPHOS, and indirectly also ROS emission [85, 86]. UCP3 contributes to the role of HKII in aerobic glycolysis. Genetic knockout or inhibition of UCP3 decreased mito-HKII and increased mitochondrial ROS emission, suggesting a role of UCP3 in HKII mitochondrial association [87]. Moreover, UCP2 in tumor cells is associated with decreased ROS emission and chemoresistance [88]. It remains to be determined if UCP2 interacts with HKII, as UCP3 does.

### B The role of mitochondrial fission/fusion in metabolic reprogramming of cancer

Mitochondria are highly dynamic organelles regulated by fission and fusion events and, as described above, cristae undergo important ultrastructural changes. Indeed, mitochondrial dynamism plays essential roles in fundamental cellular processes and metabolism, and changes in mitochondrial dynamics have been linked to cancer [89, 90]. Mitochondrial fusion is regulated by the dynamin GTPases, Mitofusin 1 and 2 (Mfn1, Mfn2) and OPA1, while mitochondrial fission is regulated by dynamin-related protein (Drp1) and recruitment/activation factors (Mff, Fis1, MiD49, and Mid50) [91]. Mitochondrial fusion is additionally correlated with altered cristae structure, enhanced assembly of respiratory super-complexes and ATP synthase assembly, often shifting cells from glycolysis to OXPHOS [92, 93]. The role of mitochondrial fission/fusion in the regulation of apoptosis and cell survival remains controversial. In OVCA cells, we observed significant higher level of mitochondrial fusion in chemoresistant cells than in their chemosensitive counterparts [83, 94]. CDDP increased significant mitochondrial fission in chemosensitive cells, but not in chemoresistant cells. Piceatannol, a metabolite of functional food compound in red wine, sensitized OVCA cells to CDDP *in vitro* and *in vivo*, by inducing dephosphorylation of mitochondrial fusion protein, Drp1 at site of Ser^637^, and promoting mitochondrial fission and apoptosis [95]. This indicates that higher proportion of tubular network in mitochondria harboring chemoresistance cells are closely related to anti-apoptosis and cell survival.

Few studies have reported the mitochondrial dynamics and energy metabolism in OVCA. Generally, through either enhanced mitochondrial fission, or decreased mitochondrial fusion, cancer cells have increased mitochondrial fragmentation, which has a functional role to promote glycolysis for energy production over OXPHOS [96]. Pro-survival factor, Survivin (BIRC5) overexpression leads to mitochondrial fragmentation through increased mitochondrial fission, shifting energy production toward glycolysis [97, 98]. Also, the inhibition of glycolysis attenuates the anti-apoptotic action of Survivin. However, cancer cells can adapt their mitochondrial structure in response to metabolic challenges similar to non-cancerous cells [96, 99, 100]. These reports demonstrate that cancer cells adapt to stressful energy conditions by modulating mitochondrial morphologies and functions generally associated with increased glycolysis.

### C Mitochondrial biogenesis and glycolysis metabolism

To maintain energy metabolism and function upon external stress, mitochondrial autophagy (mitophagy) and mitochondrial biogenesis are cooperatively required to regulate cellular homeostasis and survival [101]. Intact mitochondrial function and mitochondrial biogenesis are indeed required for survival of cancer cells [102]. Cancer cells respond to changes in glucose and other nutrient sources by altering mitochondrial biogenesis [101, 103]. Also, the key oncogenic transcription factor c-MYC (v-myc avian myelocytomatosis viral oncogene homolog) has been shown to facilitate glycolysis and mitochondrial biogenesis with associated increases in ATP production [104, 105].

### D ROS and glycolytic metabolism

Aberrant high levels of ROS generation are often associated with increase in antioxidant defenses in cancer cells [106]. ROS are potentially damaging byproducts of mitochondrial oxidative metabolism, but also function as second messengers in the transduction of extracellular signals that control cellular proliferation and cell cycle progression [107]. As a master regulator of ROS and mitochondrial energy metabolism, peroxisome proliferator-activated receptor gamma co-activator 1-alpha (PGC-1α) is partly responsible for this detoxification [15]. Through stimulating bioenergetic potential, PGC-1α promotes migration and invasion of breast cancer and facilitates chemoresistance [16]. Other transcription factors, nuclear respiratory factors (Nrf1 and Nrf2), the estrogen-related receptors (ERR-α, -β and -γ) and the nuclear factor erythroid 2-related factor 2 (Nrf2/NFE2L2) act as master regulators of anti-oxidants [108]. In spheroid OVCA cell cultures, ROS induced high expression of PGC-1α, a phenomenon associated with metastasis, stemness, and chemoresistance [109]. Also, Nrf2 is overexpressed in clear cell ovarian carcinoma exhibiting high glycolytic phenotype, and high nuclear expression of Nrf2 is associated with poor prognosis of OVCA patients [110, 111]. GSH, an important antioxidant, is critical for modulating apoptosis. In OVCA, GSH is inversely associated with chemoresistance and levels of GSH are 15-50 fold higher in chemosensitive cells than chemoresistant cells [112]. Mitochondrial GSH protects cells from ROS during mitochondrial respiration [113, 114]. Moreover, NADPH produced by the PPP is required for the generation of reduced GSH [115]. Hence, GSH functions as metabolic link between PPP and ROS balance.

## IV REGULATORY MECHANISMS OF GLYCOLYTIC METABOLISM IN OVARIAN CANCER

### A Aberrant activation of glycolysis by oncogenes

Accumulating evidence suggests that multiple tumor suppressor/oncogenes affect glucose metabolism [11, 116]. The phosphoinositide-3 kinase (PI3K)/Akt axis is a key oncogenic cell signaling pathway in cell survival and tumor progression,and may promote glycolytic reprogramming. For example, acute insulin treatment leads to the trafficking of glucose transporter in a PI3K /Akt-dependent manner [117]. PI3K is a phospholipid kinase that phosphorylates the 3′ hydroxyl (OH) group of the inositol ring of phosphoinositide lipids [118]. PI3K phosphorylates the membrane lipid phosphatidylinositol 4,5-bisphosphate (PIP_2_) to form phosphatidylinositol 3,4,5-triphosphate (PIP_3_), whereas this process is regulated by tumor suppressors phosphatase and tensin homolog (PTEN) to dephosphorylate PIP_3_ [119]. Akt is serine/threonine kinase and is highly upregulated in cancer cells [120]. Akt is activated by its recruitment to the plasma membrane by PIP_3_, followed by phosphorylation of Thr^308^ and Ser^473^ by PDK1 [121].

Akt promotes glycolysis, suppresses apoptosis, and elicits cell survival *via* multiple mechanisms [34, 122, 123]. PI3K pathway activation or mutational changes in genetic and function were common in OVCA [124]. Effect of inhibition of Akt depends on genetic heterogeneity of cancer. Inhibition of Akt1 selectively caused cancer growth in subset of OVCA cells lines, but not applied to all of OVCA subset, due to high expression of other Akt isoforms [125, 126]. Akt enhances mitochondrial HKII activity and in turn OMM stability, thereby increasing its anti-apoptotic action. Akt directly phosphorylates HKII at the consensus binding site of (RARQKT*) Thr^473^. Phosphorylated-HKII (p-HKII) is more stably associated with mitochondria, by inhibiting opening of mPTP [122, 127]. Akt facilitates binding of Bcl-xL and HKII to VDAC in OMM. Apoptosis induction and mPTP formation by Bax/Bak are prevented, largely due to pre-occupied Bax binding site by HKII for OMM permeability [35, 123]. Also, Akt activates PFK1 by the phosphorylation and activation of PFKFB2 [128]. In breast cancer, FV-429 inhibits glycolysis by attenuating Akt-mediated phosphorylation of HKII, resulting in apoptotic induction [77]. As oncogenes, KRAS and BRAF activate Akt and mutation of KRAS/BRAF enhance the expression and plasma membrane trafficking of GLUT1 [129-131]. Still, it remains unclear how the interplay of Akt/p53 affects the HKII-mediated glycolysis and other metabolic processes in cancer.

c-MYC is an oncogene and transcription factor that regulates multiple genes involved in cell proliferation, metabolism, and apoptosis [132]. Constitutive activation of c-MYC is frequently found in human cancer [133]. In OVCA, protein expression of c-MYC is higher in chemosresistant cells compared with its counterpart sensitive cells [134, 135]. High c-MYC expression is also associated with decreased overall survival and disease free survival rate [135]. c-MYC contributes to chemoresistance of cancer in part by controlling metabolism [136]. c-MYC binds and activates the promoters of key metabolic enzymes including GLUT1, HKII, PFK, and enolase 1 (ENO1), leading to an activation of the glycolysis pathway [137-139]. In addition, c-MYC cooperates with HIF-1α to promote HKII and PDKI, again shifting metabolic pathway to glycolysis [136].

mTOR is a serine/threonine kinase involved in promoting energy metabolism, cell growth, and proliferation. Elevated mTOR suppresses induction of autophagy and frequently leads to the development of tumors [34, 116]. mTOR is composed of two distinct functional complexes, mTOR complex 1 and 2 (mTORC1 and mTORC2), with their respective defining components Raptor and Rictor [51]. Akt activates mTOR, promoting anabolic metabolism and fatty acid synthesis, whereas AMPK represses mTOR, leading to catabolic energy production and fatty acid oxidation. In response to glucose deprivation, HKII can bind to mTORC1 by its TOS motif, decreasing TORC1 activity and positively regulate autophagy [140]. Under glucose depletion, inhibition of HKII is attenuated, while overexpression of HKII elevates glucose deprivation-induced autophagy [34, 140]. This indicates that cells increase their adaptability and survival by mTOR-HKII interaction under varied energy conditions.

MicroRNAs (miRNAs) are small, non-coding RNAs and post-transcriptional inhibitory regulators of gene expression. As a tumor suppressor miRNA, miR-143 downregulates HKII, whereas oncogenic miR-155 upregulates HKII expression [141]. Overall, key oncogenes promote glycolysis metabolism and cell survival, whereas tumor suppressors regulate it in OVCA, incurring its responsiveness to chemotherapy.

### B Regulation of glycolysis by p53

p53, key tumor suppressor is involved in the regulation of cellular proliferation, apoptosis, and metabolism [51]. p53 elicits its apoptotic action *via* transcription-dependent and -independent mechanisms [8]. Our lab has demonstrated that activation of functional p53 signaling is required for apoptosis in response to chemotherapy such as CDDP [6, 142]. In addition to its fundamental role in apoptosis, p53 is shown to negatively regulate glycolysis [143], as its mutation may lead to the reliance on glycolysis of cancer cells [9]. Moreover, co-deletion of p53/PTEN in prostate cancer can further increase HKII levels. PTEN deletion increase HKII mRNA translation *via* activation of the Akt-mTOR-4EBP1 pathway, whereas p53 loss enhances HKII mRNA stability by inhibiting miR-143 biogenesis [144]. p53 plays a critical role in maintaining mitochondrial integrity and function, potentially through modulating SCO2 (the synthesis of cytochrome c oxidase protein) and cytochrome oxidase II (COX II) [145, 146]. SCO2 is required for the OXPHOS, mitochondrial respiration, and the assembly of the mitochondrial complex with COX II. Therefore, depletion of p53 impairs mitochondrial structure and respiration, shifting cells toward glycolytic metabolism. p53 can also regulate the protein TP53 inducible glycolysis and apoptosis regulator (TIGAR) [147]. TIGAR has a dual function in regulating glycolysis to maintain homeostatic balance system in the cell. TIGAR leads to the accumulated production of G-6-P, preventing glycolysis. However, under hypoxic conditions, TIGAR is translocated to mitochondria and binds to HKII, decreasing ROS, promoting glycolysis, and providing cell protection [147, 148]. Mutant p53 promotes the plasma membrane trafficking of GLUT1 *via* Rho-associated protein kinase (ROCK) signalling [149]. Despite p53′s regulatory role in metabolism, detailed molecular and temporal mechanisms remain to be further elucidated.

## V TARGETING METABOLISM FOR CANCER THERAPY

### A Current status of therapeutic approaches in targeting metabolism

For the numerous aforementioned reasons, targeting glucose metabolism has been considered as a promising therapeutic strategy. However, side effects and barriers to effective treatments have led to several unsuccessful clinical trials (Table 1).

**Table 1:** Current drug development & clinical trial process for drugs targeting metabolism

Drug	Targets	Pathways	Clinical trial in cancer	Effects in cancer cells/ Clinical information	Application in OVCA
2-Deoxy glucose (2-DG)	Hexokinases	Glycolysis	Phase I discontinued due to side effects	Hyperglycemia was observed as major adverse events.	2-DG and glutaminolysis inhibitor aminooxyacetate (AOA) synergistically inhibited cell growth of OVCA [197].
3-Bromopyruvate (3-BP)			Pre-clinical	3-BP also inhibits GAPDH & LDH-A [198]	Co-treatment of 2-DG and 3-BP induced apoptosis in OVCA cells [197]. 3-BP inhibits HKII and attenuates tumor progression in OVCA [38].
Lonidamine (LND)			Phase II (Europe)	LND potentiates the chemotherapy responses in prostate, breast mouse xenograft model.	LND potentiates the responses of doxorubicin in mouse xenograft model [199]. LND and cisplatin show tolerability & activity in advanced OVCA [156].
Methyl jasmonate			Pre-Clinical	Methyl jasmonate induced apoptosis and necrosis in liver cancer [200].	Methyl jasmonate treatment in OVCA has not been conducted *in vitro* & *in vivo*.
Dichloroacetate (DCA)	PDK1	Krebs Cycle	Pre-Clinical	DCA is prescribed for lactic acidosis.	DCA & metformin together shows suppression of tumor in OVCA [160].
AT-101 (Gossypol)	LDH Bcl-xL	Bcl-xL	Phase I & II (US FDA) (Head & neck cancer)	AT-101 shows no significant efficacy when it was treated with docetaxel in head & neck cancer [201]. Clinical trial (Phase I) is on- going process in resistant leukemia & laryngeal cancer. AT-101 potentiates the responses of gefitinib in lung cancer *in vitro*.	*In vitro*, co-treatment of AT-101 and CDDP shows apoptosis in chermoresistant OVCA cells [202].
Metformin	Mitochondria Complex I	Mitochondrial Respiration	Pre-clinical (US FDA) *Recruiting patients	Metformin is prescribed for the treatment of type 2 diabetes. Metformin is effective in decrease size of solid tumor in mouse model.	Metformin induced cyclin D1 degradation & G1 cell cycle arrest in OVCA cells [166]. Currently in process of recruiting patients of OVCA for clinical trials (US FDA).

2-Deoxy-D-glucose (2-DG), Lonidamine (LND), and 3-Bromopyruvate (3-BP) are used as HK inhibitors. 2-DG, a glucose analogue, is a competitive HK inhibitor. Researches under *in vitro* and *in vivo* conditions demonstrate that concomitant use of 2-DG with inhibitors of lysosomal permeabilization increased its anti-tumor action, inducing mitochondrial damage and necrotic cell death [150]. As well, cotreatment of 2-DG with the PPARα agonist, fenofibrate (FF) leads to cancer cell death by inducing endoplasmic reticulum stress [151]. Despite these promising results, 2-DG clinical trials were stopped in phase I, since 2-DG resulted in intolerable hypoglycemia and reduced white blood cell counts in leukemia patients [152]. Also, it is toxic when given concurrently with radio therapy in glioma patients [153]. With regards to safety and efficacy, minimal concentration of 2-DG with use of concomitant chemotherapeutic agent or other glucose analog inhibitors with better specificity could be considered.

Given that HKII has variable isoforms and HKI is also highly expressed in normal cells, target specificity is of ultimate importance for safety and efficacy. Other drugs such as 3-Bromo pyruvate and methyl jasmonate, which are known to specifically detach HKII from VDAC of mitochondria, showed anti-neoplastic effects *in vitro* and *in vivo* mouse tumor models [154, 155]. These drugs have been shown to facilitate mitochondria-mediated apoptosis, whereas their targets cover a broad spectrum, including inhibition of glyceraldehyde -3-phosphate dehydrogenase (GAPDH) and LDH-A.

LND (indazole-3-carboxylic acid), inhibits HKII and aerobic glycolysis in hypoxic conditions. Since its combined therapy with temozolomide showed anti-tumor effects in brain cancer, clinical trials for combinational approaches are underway [63]. In advanced OVCA, LND has been shown to be active and tolerable in phase II clinical trial when combined with CDDP and Paclitaxel [156]. Though the results are promising, the possible application of LND in treating chemoresistant OVCA requires further investigation.

3-(3-Pyrinidyl)-1-(4-Pyrinidyl)-2-propane-1-one (3-PO) inhibits PKFB3 family of enzymes which regulate F-2,6-BP and the activity of PFK1, a rate-limiting step of glycolysis. 3-PO shows a suppressive effect in glycolytic flux and growth of cancer cells *in vitro* and *in vivo* [157]. 3-PO and its derivatives are currently in clinical trials for their efficacy and safety.

Dichloroacetate (DCA) was originally used for hereditary lactic acidosis [158, 159]; however, DCA is also considered as an anti-cancer drug that targets metabolism. DCA inhibits PDK1, thereby stimulating the activity of PDH, and shifting cells from glycolysis and lactate product to mitochondrial respiration. DCA treatment induced apoptosis in endometrioid cancer [158]. Combined treatment with DCA and Metformin showed a synergistic effect in the suppression of OVCA [160]. Also, DCA treatment with the anti-angiogenesis agent, bevacizumab (Avastin), enhanced anti-tumor effects in brain cancer through reversing hypoxic adaptation [161]. Considering that bevacizumab was recently approved (2016) for recurrent platinum-sensitive OVCA, concomitant use of bevacizumab with DCA may broaden its scope to chemoresistant OVCA [161]. Clinically, this drug has recently shown successful efficacy as durable remission for four years in glioblastoma and resistant non-Hodgkin lymphoma, but further clinical studies need to be conducted in other solid tumors in combinational therapy [162, 163].

In addition, repurposing Metformin, the first-line medication for type 2 diabetes, has gained immense interest as potential cancer treatments [164]. Metformin inhibits mitochondrial complex I and targeting mitochondrial Bcl-2 family of anti-apoptotic proteins in cancers. Metformin has also been shown to directly inhibit HKI and HKII by mimicking of its product, G-6-P as a competitive inhibitor [165]. Accumulating evidence supports its anti-tumor activity [159, 160, 166, 167]. Metformin antagonizes proliferation of cancer cells by suppressing flow of glucose- and glutamine-derived metabolic intermediates, leading to decreased citrate production and lipid biosynthesis [168]. Recent research demonstrates that its anti-tumor activity is increased when Metformin is used along with p53 stabilizers [167]. Currently, Metformin and combined chemotherapy (Cisplatin and Carboplatin) are in initial phase (recruiting patients) of clinical trial in multiple solid tumors, including OVCA [164], worldwide (USA, UK, Netherland, Australia, and Norway) [16, 169].

AT-101 (Gossypol) is used as a Bcl-xL inhibitor and is also shown to target lactate dehydrogenase. It is currently in phase I clinical trial as combined therapy with other apoptosis-inducing chemotherapy agents (Docetaxel, Cisplatin, and Carboplatin) in relapsed leukemia and laryngeal cancer. Though Imatinib (Gleevec) has never been in clinical trial covering the spectrum of targeting metabolism, it has recently been showed to also inhibit glycolysis activity [159]. Still, implementation of pre-approved drug or broadening the indications of current drugs targeting metabolism is an on-going process. Based on the outcome of previous clinical trials, specifically targeting metabolism seems justified, but the promising synergistic anti-tumor effects with conventional chemotherapy seem promising. As adjuvant chemotherapy, these strategies should be implemented to maximize synergistic effect to current chemotherapeutic agents such as Paclitaxel and Carboplatin.

In addition to chemical inhibitors of tumor metabolism, restriction of nutrition has been considered as a plausible option. The ketogenic diet (low carbohydrate, high fat and adequate protein) under certain settings may suppress tumor growth and increase chemosensitivity [170, 171]. The combination of a ketogenic diet with hyper-oxygen conditions also showed anti-tumor effects in mice [170]. However, chemotherapy frequently results in muscle wasting and cachexia, and thus patients undergoing chemotherapy are recommended to consume elevated calories and protein. In tumor bearing mice, high caloric food consumption increased survival, even in the absence of chemotherapy [172]. Still, dietary interventions in tandem with traditional therapeutics are under development and possible side effects, such as hypoglycemia, should be considered seriously [173].

### B Challenges & Strategies for targeting tumor metabolism

The major barrier to the development of these drugs is that many fail to specifically inhibit metabolic enzymes associated with cancer. Due to co-existence of multiple metabolic isoforms, many small molecule inhibitors for targeting metabolism can cause side effects since they may inhibit essential glycolytic and other metabolic pathways. Also, targeting a single metabolic step could lead to adaptive responses by another metabolic pathway, possibly resulting in side effects. Therefore, for the use of these strategies, we need to proactively investigate systematic effects of these drugs, including their toxicology and potential side effects in the kidneys or other organs.

## VI TUMOR MICROENVIRONMENT & IMMUNOMETABOLISM

### A The influence of tumor microenvironment on glycolytic metabolism

Due to rapid growth and altered metabolism, the solid tumor contains areas of hypoxia (low oxygen), increased acidification (due to high rates of glycolysis) and energy substrate limitation (due to increased distance from supporting blood vessels). Tumor cells adapt and thrive in these seemingly hostile environments by activating multiple cell survival pathways. HIFs are master transcription factors required for metabolic and survival adaptations to hypoxia [174]. Active HIF-1 is a heterodimer of an oxygen sensitive HIF-1α and constitutively expressed HIF-1β. HIF-1α stability in hypoxia allows for the transcription of many HIF-dependent genes, including multiple glycolytic genes [175, 176]. For example, HIF-1α promotes the expression of pyruvate dehydrogenase kinase (PDK) 2 and PDK4 which inactivates PDH. PDH is responsible for converting pyruvate to acetyl-CoA, which is used in TCA cycle [138]. However, excessive PDK inactivates PDH and results in the suppression of TCA cycle and OXPHOS activities, shifting the generation of ATP toward glycolysis [177]. In non-small cell lung cancer, aberrant expression of HIF-1α stimulates HKII expression, contributing to elevated glycolysis [178]. In turn, PKM2 function as an upstream effector and binds to HIF-1α, promoting glycolysis metabolism and tumorigenesis [179]. HIF-1α cooperates with c-Myc, an oncogenic transcription factor, and induces key metabolic genes, HK2 and PDK1 to promote glycolysis [136-138]. HIF-1α increases the expression of GLUT1, glucose uptake, and glucose phosphorylation [146, 180].

### B Reverse Warburg effect

The Warburg effect in the tumor microenvironment has recently been investigated from a different perspective, called reverse Warburg effect [181]. In this alternative model, epithelial cancer cells obtain energy from adjacent stromal fibroblasts, facilitating tumor growth [181]. These fibroblasts lose their stromal characteristics and are transformed into wound healing cells as a feeding source for cancer cells. In this transformed stroma, the absence of caveolin (marker for stroma) and high glycolytic enzyme expression is evident [181]. However, the specific conditions in which reverse Warburg effect occurs and how it affects tumorigenesis remain to be elucidated.

### C IL-6 (Cytokine)/CXCL14 effect on glycolysis and tumor microenvironment

In the tumor microenvironment, immunometabolism and cytokines cooperate and play critical roles in modulating the functions of immune cells and cancer cells [182, 183]. Cytokines are a family of secreted proteins that stimulate chemotaxis and cell growth. Specific proinflammatory cytokines are sometimes associated with cancer progression [184]. In OVCA patients, high level of the pro-inflammatory cytokine interleukin-6 (IL-6) is present in the serum and ascites, which is associated with poor clinical outcomes [184, 185]. Increased IL-6 and its receptor IL-6R induce invasion and metastasis of OVCA through activating the down-stream JAK-STAT3 pathway [186]. In a colon cancer mouse model, IL-6 treatment stimulated key glycolytic genes, including PFKFB3 and aerobic glycolysis, whereas the anti-IL-6R antibody decreased glycolysis [187], suggesting an important role of IL-6 in cellular metabolism.

Small cytokine, CXCL14 is a key driver for promoting metastasis of cancer-associated fibroblast (CAF) cells in breast and prostate cancer [188, 189]. CXCL14 is elevated in tumor stromal cells and promotes tumor cell proliferation and metastasis [188]. Clinically, an elevated level of CXCL14 is also correlated with poor prognosis [190]. In OVCA, CXCL14 in CAF mediates delivery of long non-coding RNA, LINC00092, a driver of metastasis [191]. Mechanistically, CAFs with high CXCL-14 promotes LINC00092 in OVCA. LINC00092 binds to PFKFB2, promoting cancer metastasis [191]. Chemokine growth-regulated oncogene (Gro)1 functions as an inducer of senescence in fibroblast, leading to malignant transformation of ovarian epithelial cells [192]. This suggests that Gro1 may be involved in the reverse Warburg effect, with adjacent fibroblasts providing nutrients to the cancer cells. Precisely how multiple cytokines interplay in the tumor microenvironment and regulate cellular metabolism remains to be determined.

Infiltrating T cells compete with tumor cells for energy substrates as both cell types consume copious energy. Incidentally, glucose deprivation of CD8+ T cells in the tumor microenvironment decreases its cytotoxic action on tumor cells [11, 193]. Conversely, CD8+ T cells abolish this effect by altering GSH and cysteine metabolism in fibroblasts, suggesting a critical role of CD8+ T cells in chemosensitivity in tumor microenvironment [194]. Targeting glycolysis in cancer cells, but not T-cells, is therefore an important consideration when manipulating metabolism in the tumor microenvironment.

### D Influence of exosome delivery on tumor metabolism

Exosomes are nano-sized vesicles derived from endocytic compartments and released by multiple cell types, including cancer cells. Accumulating evidence suggests that purified exosomes contain functional miRNA and small RNA, and long non-coding RNA [195]. Still, it remains unclear whether and how exosomes affect tumor microenvironment and tumor metabolism.

Nonetheless, using exosomes, cancer cell-derived miR-122 has been transferred to normal cells in premetastatic niches, thereby suppressing glucose uptake and utilization by down-regulating PK. Using glucose source derived from these niche cells, cancer cells facilitate the massive energy needs during metastatic growth [196]. This metabolic programming of cancer facilitates metastasis to other organs. Hence, cancer cell-derived extracellular miR-122 can reprogram systemic energy metabolism to facilitate disease progression and metastasis of breast cancer [196].

## VII SUMMARY & FUTURE DIRECTIONS

Elevated metabolism is a key characteristic of multiple cancers. Many key glycolytic enzymes, including HKII, PFK, and PKM2 are elevated in tumor cells, and are involved in anti-apoptotic and cell survival mechanisms associated with chemoresistance. Considering that these enzymes are regulated by oncogenes (e.g., Akt, mTOR) and tumor suppressors (e.g., p53), it is likely that defective control of tumor suppressors may lead to dysregulated metabolism and growth of cancer cells. In addition, the tumor microenvironment contributes to elevated metabolism in cancer cells. In this context, targeting dysregulated metabolism may be an effective strategy to suppress tumor growth. However, recent clinical trials have demonstrated that targeting metabolism alone is not sufficient for cancer therapy. Instead, adjuvant chemotherapy in combination with targeting tumor metabolism may be the optimal therapeutic strategy, especially in overcoming chemoresistant OVCA. With continued emphasis on the design of specific inhibitors of tumor metabolism, the application of combined therapies is likely to improve anti-cancer strategies.
